# Congress of Physicians—Forty-First Anniversary of the Georgia Medical Association

**Published:** 1890-05

**Authors:** 


### CONGRESS OF PHYSICIANS.—FORTY-FIRST ANNIVERSARY OF THE GEORGIA MEDICAL ASSOCIATION.

Representative men of the profession from all sections of Georgia meet in the city of Brunswick, and for three days (16th to 18th April inclusive) confer one with another as to the great principles of medicine and surgery. Old ideas were revived and discussed, new ones advanced and ventilated. Actuated by a common desire and a unity of purpose, the energies of the whole assembly, as of one man, were bent towards the upbuilding of the Association and the advancement of the medical science. The meeting was a full one, and the number of accessions to the role of membership greater than for any year in the last decade.

The programme was as follows: April 16, 10:30 A.M.—Dr. J. S. Todd, of Atlanta, the retiring President, called the meeting to order. After a few remarks in leave-taking, he turned the meeting over to Dr. R. O. Engram, of Montezuma, Vice-President, who presided in the absence of Dr. J. B. S. Holmes, of Rome, President-elect. The session was opened with prayer by Rev. E. Z. F. Golden.

An address of welcome on the part of the physicians of Brunswick to the Association was now made by Mr. W. E. Kay, in the absence of Dr. H. Burford, who should have delivered the address.

Mr. J. W. Bennett now delivered an address of welcome in behalf of the city of Brunswick. Both of these addresses were full of welcome and kind greetings. The response, on the part of the Association, was to have been made by Dr. G. W. Mulligan, of Washington, Ga., but he was not present, owing to some failure in the railroad connections, and Dr. Eugene Foster, of Augusta, was called on, and responded in some impromptu remarks.

The President's address was next in order. In his absence, the Vice-President, Dr. R. O. Engram, read a paper on "Food, in Health and Disease."

The Committee on Programme now reported, through Dr. Foster, its chairman. Report was adopted.

Board of Censors was next announced as consisting of Drs. R. Battey, Rome; S. B. Hawkins, Americus; M. H. O'Daniel, Milledgeville; B. R. Doster, Blakely, and G. W. Mulligan, Washington. In the absence of Drs. Battey and Hawkins, the President appointed Drs. Griggs and Todd to act on the Board of Censors in their stead.

At this juncture, by virtue of a motion of Dr. Todd, a dispatch was sent to Dr. Holmes conveying the sympathy, confidence and continued love of the Medical Association of Georgia for him in this, his hour of trial.

After the reading of letters from Drs. Holmes, Hawkins, Cortelyou, Humphries, Wells and Nunn, expressing regret at not being able to attend the meeting of the Association, the President declared the Association adjourned, to meet at 9 A. M., April 17.

During the afternoon we were the willing captives of the citizens of Brunswick. They had arranged for an excursion around the harbor on the Pope Catlin. About 2 P. M. two hundred of the citizens in company with the visiting physicians boarded the steamer for Ocean Pier, St. Simons. Excellent music was furnished by the Atlantic band, and refreshments were served on the boat; but a rare treat awaited us on the island, viz., an oyster roast and clam bake. After partaking heartily of these, we rode over the island on the street car, and then to the boat and back to Brunswick.

April 17, 9 A. M.—Association was called to order by Dr. Engram, Vice-President.

A motion was passed authorizing the Secretary to furnish a copy of Dr. Engram's paper on "Food, in Health and Disease" for the Brunswick *Times* for publication.

Dr. Virgil O. Hardon, of Atlanta, next favored the Association with a paper on "Two Cases of Rare Ovarian Tumors," which appears in this JOURNAL.

"Aseptic *vs.* Antiseptic Surgery" was the subject of the next paper, read by Dr. Samuel C. Benedict, of Athens. Dr. Benedict is a strong advocate of both aseptic and antiseptic surgery. He did not oppose the two, as one might suppose from the reading of the subject, but classed under the head of aseptic surgery all cases treated without medicaments or germicides, and under the head of antiseptic surgery such as were treated with these agents.

An animated discussion followed. Dr. J. A. Dunwoody, of Brunswick, expressed his appreciation of the paper, and concurrence in the opinions expressed.

Dr. W. F. Westmoreland, Jr., expressed the belief that asepsis and antisepsis have done more for surgery than any one agent, anæsthesia not excepted. They are really principles of surgery —said that antiseptic surgery could be practiced successfully where you could not rely on asepsis, viz., in hospitals and hovels. His arguments in favor of antiseptic surgery were, less cost, less time, completer union, less pain after operation.

Dr. J. F. Lancaster, of Flovilla, endorsed the views of Dr. Benedict; thought to get the most perfect results we should practice both asepsis and antisepsis.

Dr. J. McF. Gaston, of Atlanta, said he thought the views presented by the author and the views of those who had participated in the discussion very appropriate, but the paper should not have been limited to asepsis and antisepsis, but should have extended to septic surgery. The most important phase of the treatment of wounds is the prevention of trouble from the undue use of applications which may aggravate the existing condition, and a caution is needed against septic germicides. The crying evil connected with the surgery of to-day is the result of measures which are classed as antiseptics, in such a way as to contaminate the structures, and lead to serious constitutional troubles. He thought all operations on normal tissues and conditions not involving microbes might be conducted upon the principle of asepsis. He would avoid the use of corrosive sublimate in abdominal surgery, chloride of sodium answering all the conditions and attended with no risk. He would also avoid bringing a solution of corro. sublimate in contact with freshly cut absorbent surface. Where sepsis already exists, it should be corrected by germicides, but with due regard to safety of patient.

Dr. Eugene Foster, of Augusta, said he thought cleanliness the most fruitful source of success in surgery. He didn't have the faith in antiseptics as expressed by some of its advocates. Referred to Tait, one of the greatest living surgeons, as not using antiseptics.

Dr. J. S. Todd, of Atlanta, closed the discussion, stating that he was the first to urge the use of antiseptics in surgery in the Atlanta Medical College. Referred to the antiseptic properties of turpentine. Said it was a fine germicide and urged its use in typhoid fever upon that principle.

Dr. H. McHatton, of Macon, read a paper on "Railroad Surgery." He reported 170 cases of railroad surgery, classifying the various wounds and giving treatment for same.

In the discussion Dr. W. H. Elliott spoke of the advantage of plaster splint or plaster bandage. He objected to plaster bandage because it would not give or adapt itself if swelling occurs. He recommended a flannel bandage covered with a coat of plaster, opened the whole length of splint so as to allow it to adjust itself to parts if swelling occurs.

Dr. A. P. Taylor thought it was safer to operate after shock consequent upon injury.

Dr. P. L. Hillsman favored the view of Dr. Taylor as regards time of operating. Said he had found a dressing of oxide zinc and glue on flannel very serviceable. Shellac on flannel also made a convenient dressing.

Dr. Westmoreland advocated the immediate use of plaster dressing, if there was not much bruising of parts and if patient was seen before swelling began. Said swelling was frequently due to mobility of bones and plaster would prevent this. Thought it best to operate during shock if it was not too severe, as the injury and operation would be but one continuous shock, whereas the patient would be subjected to an additional shock if operation was delayed. Favored warm douche during operation, as it lessened shock. Said plaster was very porous and so less liable to cause excoriations of skin than other dressings.

Following the programme Dr. Westmoreland next made an oration on the " Surgery of the Past, Present and Future."

Letters were now read from Drs. A. C. Davidson, T. M. Holmes, W. D. Bizzell and R. C. Word, expressing regrets at not being able to attend the Association.

3:30 P. M.—D. L. Bullard read a paper on "Ostio Sarcoma," exhibiting patient. Paper will be published in this JOURNAL.

Dr. Gaston was called on to examine patient and found the tumor movable to a limited extent; adhesions firmer on the inner than on the outer portion of frontal bone. The nodular irregularity of superficial portion had greater density in some parts than others, and the purplish discoloration and enlargement of cutaneous veins lead to the suspicion that the tumor is encephaloid. Referred to the malignancy of such disease and proneness to reproduction when removed. It had extended to orbit and by pressure on optic nerve destroyed sight. If diagnosis of fibro-sarcoma should be verified there would not be so much danger of redevelopment. In view of rapid growth of tumor he recommended operation for its removal, allowing large denuded surface to heal by granulation.

Dr. K. P. Moore now read a paper on "The Female Urethra, a Source of Trouble Liable to be Overlooked in our Gynecological Investigations." This paper will appear in the JOURNAL.

In discussion Dr. J. G. Earnest said his views accorded with those just expressed; emphasized the importance of a thorough examination of urethra, to dilate it if necessary. Recommended thorough excision as the only treatment for caruncles.

Dr. Virgil O. Hardon, while he thought excision the proper treatment for caruncles, warned against cutting into cellular tissue, as it often leads to cicatricial tissue, which will be followed by a very unpleasant train of symptoms. Said dilatation of urethra for examination should be done very cautiously, as it sometimes leads to incontinence. Spoke of the simplicity and efficacy of Emmett's button-hole operation for chronic urethritis. Said diseases of urethra were not very common. Blame was often referred to urethra when its true source was to be found with the uterus.

Dr. Moore next read a paper prepared by Dr. A. C. Davidson, of Sharon, on "A Unique Case of Senile Gangrene; followed by a Bloodless Operation without Tourniquet or Ligature."

Drs. Carter and Westmoreland discussed the paper.

Dr. McHatton now read a paper written by Dr. H. J. Williams, of Macon, on "The Importance of Microscopical and Chemical Analysis of the Urine." In discussion Dr. McHatton spoke of the importance of feeding in kidney affections. Gives no medicine except when there is not sufficient excretion of urea.

Dr. Todd said in all cases of senile gangrene seen by him the urine contained sugar. He also found sugar in urine of seven out of eight patients treated for carbuncle past middle life. Spoke of the efficacy of pilocarpine and sweat baths as diuretics. Dr. A. W. Griggs spoke of the value of chloroform in acute albuminuria—dose gtt. xii increased to gtt. xx every five hours. Dr. J. W. Duncan said he had found chloroform a valuable agent in albuminuria and wished to corroborate the remarks of Dr. Griggs.

Thursday evening the citizens and physicians were made better acquainted around the banqueting board. Good cheer prevailed and the hours passed swiftly by.

Friday morning, 9:30.—Association was called to order by Dr. Todd, in the absence of Dr. Engram.

President announced that Dr. Holmes had appointed Dr. Lindsay Johnson, of Cartersville, as the next annual orator.

The following were appointed delegates to the American Medical Association: Drs. Todd, Moore, Hopkins, O'Daniel, Foster, Whitehead, McIntosh, Dugas, Lane, Calhoun, Hardon, Stone, Duncan, Dunwoody, Butts, Hawkins, Powell, Heard, Goodrich, Cortelyou, Greene, Holmes, Battey, Campbell, Lancaster, Engram, Hall, Ford, Tate, Dobbins, Kelly and Gaston.

Auditing Committee reported favorably on the reports of Secretary and Treasurer.

Dr. R. O. Cotter next read a paper on "Notes on the Progress of Opthalmology and Rhinology." In discussion Dr. Calhoun spoke of the advantage of antisepsis in operations about the eye—said he had made 135 cataract operations without a single bad result, and he thought the good results due largely to antisepsis.

A letter was read from the Missouri State Medical Association, sending fraternal greetings to the Georgia State Medical Association, and asking that doctors be sent from the Georgia Association to the meeting of Missouri doctors, to be held May 6, 7 and 8, at Excelsior Springs, Mo. Secretary was ordered to acknowledge receipt of letter, and to extend like invitation to Missouri Medical Association.

Nominating Committee made following report, which was adopted: President, Dr. A. W. Griggs; first Vice-President, Dr. J. A. Dunwoody; second Vice-President, Dr. E. W. Lane; Censor, Dr. H. McHatton. Place of meeting, Augusta.

Dr. J. A. Butts favored the Association with a few remarks on "The Climatology of Brunswick," claiming that it was anti-tubercular, anti-rheumatic and anti-malarial. In discussion, Dr. Todd spoke of the benefit of the climate to asthmatics. Dr. Hopkins indorsed the statements made by Dr. Butts.

Secretary now read a paper of Dr. R. O. Engram's on "Strictures of the Male Urethra and Forms of Neuroses."

Dr. Gaston in discussion said the paper furnished some new ideas to him, among others, the intricate relations between urethral troubles and skin lesions. Said he didn't agree with the author in attributing varicocele to urethral troubles. Varicocele often exists where there is no concomitant of this nature, and no instances are cited where relief of stricture was followed by relief of varicocele. The ataxic phenomena said to be relieved by dilatation of urethra are not conclusively demonstrated. He recognized the efficacy of introducing metallic sound for seminal emissions, but deprecated the cutting and all violent dilating of urethra in treatment of seminal weakness. Didn't believe in dilatation after internal urethrotomy. Pass sound after operation to see if stricture is cut and then let it rest for ten days.

Committee to petition the General Assembly for a State Board of Health reported through Dr. Foster, its chairman. He said the committee did all they could but failed and now asked to be discharged. Discharge granted.

President continued same committees on programme, publication and prize essays.

On motion of Dr. Gaston, an honorarium of $100 each was tendered to the Secretary and Treasurer.

On motion of Dr. Foster, a committee was appointed to be known as Committee on Membership. This committee will be expected to correspond with all physicians in the State not members of the Association and ascertain why they are not members, and in every possible way enlist the interest of the profession in the Association.

Dr. A. W. Griggs, the newly elected President, was now introduced by Dr. Todd, and in taking his seat thanked the Association for the confidence reposed in him by calling him to the President's chair.

After the passage of resolutions of thanks to the citizens of Brunswick for their hospitality during our stay in their midst, the Association adjourned to meet in Augusta, the third Wednesday in April, 1891.

Dr. J. McF. Gaston availed himself of the privilege afforded by the President to make the following statement. A gentleman about 32 years old was attended in consultation during the summer of '88, who presented symptoms of acute psoas abscess with high fever for several days. The pain was limited to the right iliac region, and right lumbar region. After a time there developed a well marked prominence in front of right kidney and extending forward above the crest of the ilium into the iliac region. There was in the meantime febrile excitement alternating with chilliness and great tenderness upon pressure, with a sense of deep fluctuation on palpation over the prominence. With these indications of a psoas abscess, it was thought proper at the expiration of a week from my first visit to make an opening for its evacuation. But upon going on the following day with two colleagues and a brother of the patient, it was found that the prominence had disappeared and there was no evidence of fluctuation where it had previously existed.

Instead of making the incision as intended, an exploratory puncture was made at the most salient aspect of the right lumbar region about midway between the false ribs and crest of the ilium. It passed through a dense membrane into a cavity without any discharge by suction, and it was then evident that the abscess had found an outlet into the colon where it lies in contact with the muscles. On the following morning there were free evacuations from the bowels, as there had been frequently during the previous week; yet it was stated that a physician, who was called to the case subsequently, treated the patient for fæcal impaction and attributed the previous disorder to constipation of the bowels. The patient afterwards returned to Jacksonville, Fla., where he was under treatment of Dr. R. B. Burroughs for local trouble in the lumbar and iliac regions. He being unable to attend to his duties, returned to his home in Illinois and sought the services of Dr. C. T. Parks, of Chicago, who made an incision on the dorsum of the right ilium about two inches below the crest. This entirely closed and a swelling occurred between the cicatrix and the sacrum, which pointed and broke spontaneously into the line of the incision, from which a purulent discharge has continued for several weeks up to this time. The examination with a probe, this morning in the presence of several colleagues, revealed a fistulous track extending from near the sacro-iliac symphysis, and the opinion expressed by those present was that the pus resulted from some carious bone in that vicinity calling for an operation. With a view to elicit the judgment of others having experience in similar cases, any members of the Association who may be interested in this matter are invited to examine the patient after close of morning session. Dr. Gaston reports a corroboration of the previous opinion by those who made the subsequent examination. L. P. K.

